# Three-Dimensional Printing and CAD/CAM Milling in Prosthodontics: A Scoping Review of Key Metrics Towards Future Perspectives

**DOI:** 10.3390/jcm14144837

**Published:** 2025-07-08

**Authors:** Catalina Cioloca Holban, Monica Tatarciuc, Anca Mihaela Vitalariu, Roxana-Ionela Vasluianu, Magda Antohe, Diana Antonela Diaconu, Ovidiu Stamatin, Ana Maria Dima

**Affiliations:** 1 Department of Dental Prosthesis Technology, Faculty of Dental Medicine, “Grigore T. Popa” University of Medicine and Pharmacy, 700115 Iasi, Romaniatatarciucm@yahoo.com (M.T.); antonela.diaconu@umfiasi.ro (D.A.D.); 2 Department of Prosthodontics, Faculty of Dental Medicine, “Grigore T. Popa” University of Medicine and Pharmacy, 700115 Iasi, Romaniaovidiu.stamatin@umfiasi.ro (O.S.); 3 Independent Researcher, 700115 Iasi, Romania

**Keywords:** prosthodontics, additive manufacturing, 3D printing, subtractive manufacturing, CAD/CAM milling, accuracy, efficiency, workflow, material, trueness

## Abstract

**Background/Objectives**: Digital prosthodontics increasingly utilize both additive (3D printing) and subtractive Computer-Aided Design/Computer-Aided Manufacturing (CAD/CAM), yet comprehensive comparisons remain limited. This scoping review evaluates their relative performance across prosthodontic applications. **Methods**: Systematic searches (PubMed, Scopus, Web of Science, Embase, 2015–2025) identified 28 studies (27 in vitro, 1 retrospective). Data were extracted on accuracy, efficiency, materials, and outcomes. **Results**: CAD/CAM milling demonstrated superior accuracy for fixed prostheses, with marginal gaps for milled zirconia (123.89 ± 56.89 µm), comparable to optimized 3D-printed interim crowns (123.87 ± 67.42 µm, *p* = 0.760). For removable prostheses, milled denture bases achieved a trueness of 65 ± 6 µm, while SLA-printed dentures post-processed at 40 °C for 30 min showed the lowest root mean square error (RMSE) (30 min/40 °C group). Three-dimensional printing excelled in material efficiency (<5% waste vs. milling > 30–40%) and complex geometries, such as hollow-pontic fixed dental prostheses (FDPs) (2.0 mm wall thickness reduced gaps by 33%). Build orientation (45° for crowns, 30–45° for veneers) and post-processing protocols significantly influenced accuracy. Milled resins exhibited superior color stability (ΔE00: 1.2 ± 0.3 vs. 3D-printed: 4.5 ± 1.1, *p* < 0.05), while 3D-printed Co-Cr frameworks (SLM) showed marginal fits of 8.4 ± 3.2 µm, surpassing milling (130.3 ± 13.8 µm). Digital workflows reduced chairside time by 29% (154.31 ± 13.19 min vs. 218.00 ± 20.75 min). All methods met clinical thresholds (<120 µm gaps). **Conclusions**: Milling remains preferred for high-precision fixed prostheses, while 3D printing offers advantages in material efficiency, complex designs, and removable applications. Critical gaps include long-term clinical data and standardized protocols. Future research should prioritize hybrid workflows, advanced materials, and AI-driven optimization to bridge technical and clinical gaps.

## 1. Introduction

The evolution of prosthodontics from analog craftsmanship to digital precision marks one of the most significant advancements in modern dentistry [1,2]. Traditional prosthetic fabrication, long dependent on manual labor, impression materials, and lost-wax techniques, is increasingly being replaced by computer-aided design and computer-aided manufacturing (CAD/CAM) and 3D printing technologies that promise greater accuracy, efficiency, and reproducibility [3,4]. This shift is not merely technical but represents a fundamental change in treatment workflows, patient outcomes, and the economic landscape of dental care. However, as digital workflows become mainstream, critical questions remain unanswered: Which method—subtractive milling or additive 3D printing—delivers superior clinical performance? And how should clinicians, researchers, and manufacturers navigate the rapidly expanding array of materials and technologies?

CAD/CAM technology, introduced in the 1980s, has matured into a gold standard for producing high-strength, aesthetically precise dental restorations [5]. By milling pre-fabricated blocks of zirconia, lithium disilicate, or polymethyl methacrylate (PMMA), CAD/CAM milling systems enable same-day prostheses with well-documented longevity and marginal fit [6,7]. In contrast, 3D printing, encompassing stereolithography (SLA), digital light processing (DLP), and selective laser melting (SLM), has emerged as a disruptive force, particularly for applications requiring complex geometries, such as surgical guides, removable dentures, and temporary crowns [8,9,10]. Advocates highlight its material efficiency, customization potential, and ability to produce structures that milling cannot replicate [11,12].

Yet, the rapid adoption of these technologies has outpaced rigorous comparative research. Systematic reviews to date have largely examined CAD/CAM milling and 3D printing in isolation, often focusing narrowly on fixed prostheses or specific materials [13,14,15]. Few studies provide a holistic assessment of accuracy (trueness and precision), clinical efficiency (production time and cost), and long-term performance across different prosthetic applications [16]. Moreover, conflicting findings persist: some studies suggest that milled prosthesis exhibit superior marginal adaptation, while others argue that 3D printing achieves comparable accuracy with greater design flexibility [17,18,19,20]. These discrepancies may stem from methodological inconsistencies, such as variations in scanning technologies, measurement protocols, or post-processing techniques.

Despite their widespread adoption, a critical gap persists in the literature: a comprehensive comparison of their accuracy, efficiency, and clinical applicability across diverse prosthodontic applications. How does the trueness and precision of 3D-printed prostheses compare to milled counterparts? Which method offers superior efficiency in terms of speed, cost, and material utilization in different scenarios? And where should future innovations concentrate to overcome current limitations in materials, standardization, and long-term performance?

This scoping review seeks to map the evolving landscape of digital prosthodontics by synthesizing the most recent evidence on additive and subtractive manufacturing, addressing two main questions:

1. What is the latest evidence on the accuracy, efficiency, and material behavior in 3D printing and CAD/CAM milling for dentures?
2. Where should future innovation focus?

## 2. Methods

### 2.1. Design

This scoping review was conducted following the “PRISMA Extension for Scoping Reviews (PRISMA-ScR): Checklist and Explanation”; the completed checklist is provided in Supplementary File S2 [21].

### 2.2. Aim

The aim of this study is to benchmark the performance of 3D printing and CAD/CAM milling in both fixed and removable dentures, guiding future innovation priorities.

### 2.3. Search Strategy

To ensure a comprehensive and methodical approach, this scoping review employed a systematic search strategy across four major databases: PubMed, Scopus, Web of Science, and Embase, spanning the last decade, from January 2015 to February 2025 (Supplementary File S1). This timeframe was selected to capture the rapid evolution of digital prosthodontics, including recent advancements in materials, workflows, and manufacturing technologies. The search strategy was designed to balance sensitivity and specificity, combining controlled vocabulary (MeSH terms) with free-text keywords to retrieve the relevant literature while minimizing irrelevant results.

As a genuine scoping review, this paper prioritized breadth over specificity, which is why it did not rigidly follow PICO (designed for systematic reviews). Instead, it employed a flexible, comprehensive search strategy structured around three core conceptual domains, to capture all relevant literature on digital prosthodontic fabrication methods:

1. Population/Application
  - (“prosthodontics” OR “dental prosthesis” OR “crown” OR “bridge” OR “denture” OR “implant prosthesis”).
2. Interventions
  - (“3D printing” OR “additive manufacturing” OR “stereolithography” OR “digital light processing” OR “fused deposition modeling”);
  - (“CAD/CAM” OR “computer-aided design” OR “subtractive manufacturing” OR “milling” OR “CNC machining”).
3. Outcomes
  - (“accuracy” OR “precision” OR “trueness” OR “marginal fit” OR “dimensional error”);
  - (“efficiency” OR “production time” OR “workflow” OR “cost-effectiveness”);
  - (“material properties” OR “mechanical strength” OR “biocompatibility” OR “longevity”).

To refine the search, filters were applied to include only peer-reviewed articles, clinical trials, in vitro studies, and systematic reviews published in English. Case reports, editorials, and non-comparative studies were excluded to maintain focus on robust, evidence-based research. The full search syntax, including database-specific adaptations, is detailed in the Supplementary Materials. This rigorous methodology ensures that the review captures the most relevant and high-quality evidence while providing transparency for reproducibility. By casting a wide yet targeted net across multiple databases, this search strategy aims to synthesize the current state of knowledge while identifying gaps that warrant further investigation.

### 2.4. Eligibility Criteria

The review was limited to articles published in the last ten years and in English. Specifically, the paper included peer-reviewed comparative studies that evaluated additive and subtractive manufacturing methods. Systematic reviews and meta-analyses were considered for background context and reference mining, though they did not constitute primary data for this comparative analysis. To ensure meaningful comparisons, included studies had to report measurable outcomes such as marginal gap measurements, trueness/precision analyses, production time metrics, workflow efficiency assessments, and material property evaluations (e.g., flexural strength, wear resistance). The scope encompassed all major prosthodontic applications, from single-unit crowns and multi-unit bridges to complete dentures and implant-related components like surgical guides and custom abutments. Table 1 outlines eligibility criteria with rationales.

### 2.5. Source Selection Process

The source selection process was documented using the PRISMA-ScR protocol flow diagram [21]. The evidence identification process for this scoping review followed a rigorous, multi-stage screening methodology to ensure comprehensive coverage of the relevant literature while maintaining methodological precision. The initial database searches yielded 1141 records, representing the broad scientific interest in digital prosthodontic technologies. To enhance the efficiency of subsequent screening, database-specific filters were first applied, excluding 246 records that did not meet basic criteria such as publication type or date range.

A significant deduplication phase followed, where 304 redundant records were systematically removed through a two-tiered verification process. Initial automated removal was performed using Zotero’s duplicate detection supplemented by manual verification, followed by a more stringent, Microsoft Excel-based deduplication employing a customized formula to ensure absolute precision in record management.

Title and abstract screening of the remaining 561 records was conducted with adherence to the inclusion framework. A total of 526 records were excluded, which (1) presented non-comparative data; (2) addressed irrelevant applications (e.g., orthodontic devices or non-dental manufacturing); or (3) were published in languages other than English. This refined the pool to 35 potentially eligible studies warranting full-text assessment.

During full-text retrieval, one study proved inaccessible. The remaining 34 studies underwent rigorous eligibility evaluation. Two reviewers, AMD and RIV, independently screened each study, resolving discrepancies through consensus. Six records were excluded for specific limitations: one economic analysis (lacking technical performance metrics), four systematic reviews (retained only for contextual reference), and one clinical report (without quantitative outcome data).

### 2.6. Critical Appraisal of Sources

Since this study follows a scoping review approach, it does not include a formal systematic evaluation with critical assessment. Nonetheless, an in-depth analysis of the available data was carried out to meet the review’s goal.

## 3. Results

### 3.1. Selection of Studies

**Figure 1:**
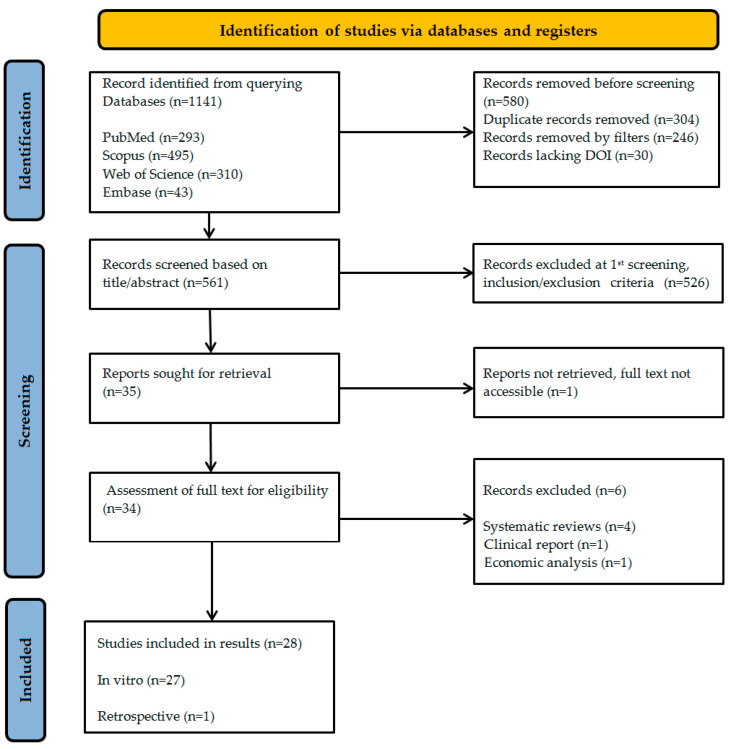
Flow diagram of the study selection process according to PRISMA ScR guidelines.

The scoping search yielded 1141 records from four databases. After filtering and removing duplicates, 561 studies underwent title/abstract screening. Following PRISMA ScR guidelines, 34 full-text articles were assessed for eligibility, with 28 studies meeting the inclusion criteria (Figure 1).

### 3.2. Overview of Selected Studies

The 28 studies that met the inclusion criteria included 27 in vitro studies (of which, one was a non-randomized comparative laboratory study [22,23,24,25,26,27,28,29,30,31]) and one retrospective cohort study [32]. This resulted in a final cohort of methodologically heterogeneous studies (Table 2).

**Figure 2:**
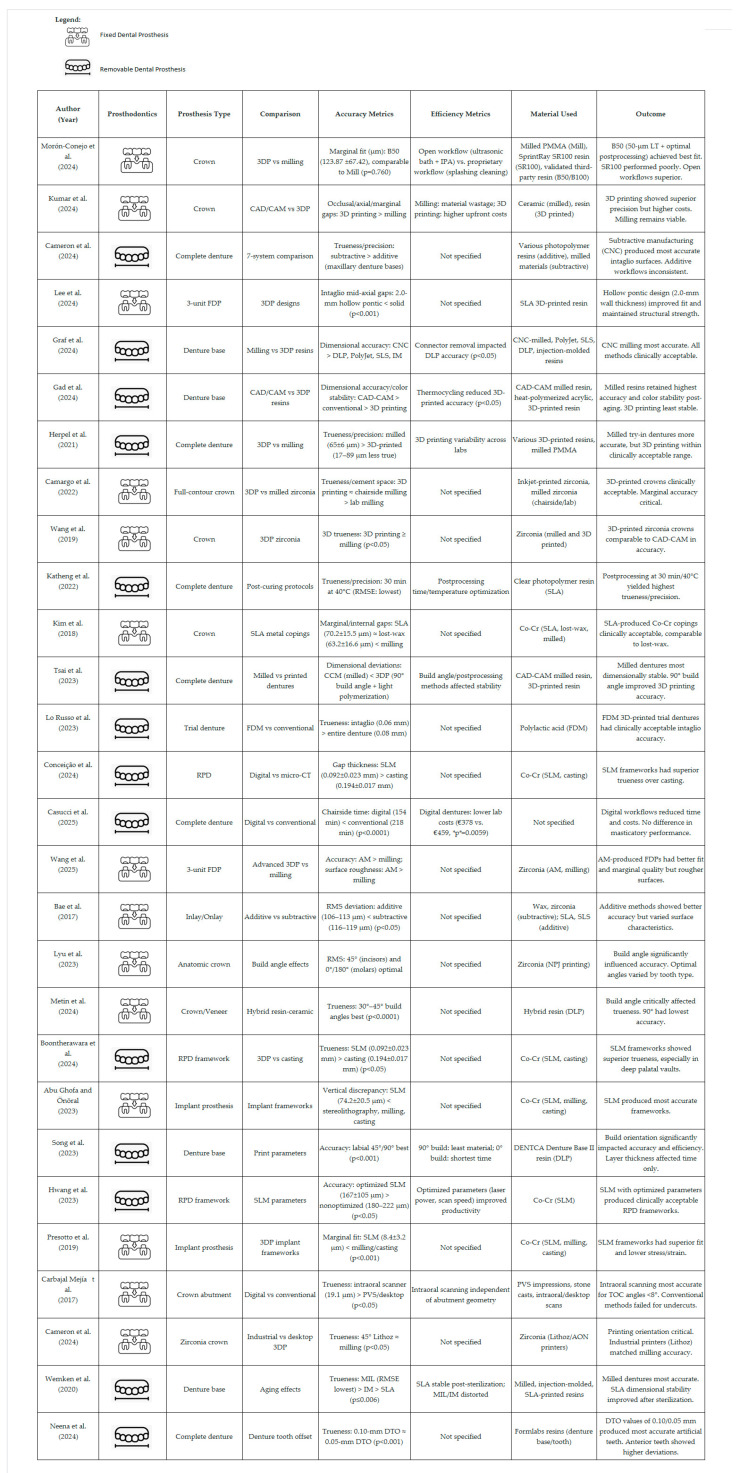
Selected studies with data description, core metrics, and outcome [22,23,24,25,26,27,28,29,30,31,32,33,34,35,36,37,38,39,40,41,42,43,44,45,46,47,48,49].

The majority of the evidence comes from in vitro studies (n = 27), but the incorporation of retrospective and multicenter data offers a practical perspective on the study’s objectives. Unlike systematic reviews, which focus on answering specific research questions, scoping reviews seek to comprehensively chart all existing evidence, encompassing a variety of study designs. The data description of the selected studies is presented in Figure 2.

### 3.3. Summary of Results: Evidence of CAD/CAM Milling and 3D Printing in Prosthodontics

The evaluation of the 28 studies revealed a balanced mix between fixed and removable dentures, together with critical insights into the performance of additive (3D printing) and subtractive (milling) manufacturing across prosthodontic applications, including crowns, bridges, removable dentures, and implant-supported prostheses. The findings are presented thematically, addressing accuracy, efficiency, and material behavior, to identify key outcomes that may guide clinical considerations.

### 3.4. Accuracy: Trueness, Precision, and Marginal/Internal Fit

Accuracy is paramount in prosthodontic restorations, influencing longevity and patient satisfaction. Studies indicate that both milling and 3D printing can achieve clinically acceptable fit, though outcomes vary by technique, material, and postprocessing.

- Interim Crowns: Morón-Conejo et al. found that DLP-printed interim crowns with 50 µm layer thickness and ultrasonic postprocessing (123.87 ± 67.42 µm) matched milling’s marginal fit (*p* = 0.760), while SprintRay’s proprietary resin and workflow with a 100 µm layer thickness (SR100) underperformed (*p* < 0.001) [33]. SR100 exemplifies a trade-off in proprietary 3D printing systems: while they offer convenience, they may sacrifice accuracy due to rigid workflows and suboptimal postprocessing. The study advocates for validated open workflows for better clinical outcomes. Kumar et al. reported superior precision in 3D-printed crowns, with smaller occlusal, axial, and marginal gaps than milled counterparts [34].
- Fixed Prostheses: Wang et al. observed that 3D-printed zirconia crowns exhibited comparable or better trueness than milled ones across external, intaglio, and marginal surfaces (*p* < 0.05) [40]. Similarly, Camargo et al. noted that inkjet-printed zirconia crowns had intermediate accuracy between chairside and industrial milling, with clinically acceptable cement-space thickness [39].
- Removable Dentures: Cameron et al. demonstrated that milled denture bases had the truest intaglio surfaces, while 3D-printed bases showed inconsistency across systems [35]. Graf et al. confirmed CNC milling’s superiority in dimensional accuracy over PolyJet, SLS, and DLP (*p* < 0.001), though all methods were clinically viable [37].

### 3.5. Efficiency: Balancing Speed, Cost, and Waste

Efficiency encompasses production speed, material waste, and labor intensity. Digital workflows generally reduce chairside time, but outcomes depend on technology and design parameters.

- Chairside vs. Laboratory Production: Casucci et al. reported digital dentures reduced chairside time by 30% (154.31 ± 13.19 min vs. 218.00 ± 20.75 min, *p* < 0.0001) and lowered laboratory costs [32].
- Build Orientation and Layer Thickness: Song et al. found 90° build angles minimized material use but increased printing time, whereas 45° orientations improved accuracy (*p* < 0.001) [46]. Metin et al. noted 30–45° angles optimized trueness for crowns and veneers (*p* < 0.0001) [44].
- Post-processing Impact: Katheng et al. highlighted that SLA dentures postpolymerized at 40 °C for 30 min achieved the highest trueness and precision (*p* < 0.001) [41].

### 3.6. Material Performance

Material properties influence restoration durability, aesthetics, and biocompatibility. Milling and 3D printing exhibit distinct advantages based on material selection.

- Mechanical Performance: Lee et al. showed hollow pontic designs (2.0 mm wall thickness) in SLA-printed FDPs improved axial fit without compromising strength (*p* < 0.001) [36]. Kim et al. noted SLA-assisted cast Co-Cr copings had marginal discrepancies (70.2 ± 15.5 µm) comparable to lost-wax casting (63.2 ± 16.6 µm), outperforming milled copings (130.3 ± 13.8 µm, *p* < 0.05) [42].
- Color and Dimensional Stability: Gad et al. found milled denture bases had superior color stability (lowest ΔE00) and dimensional accuracy after aging, while 3D-printed resins degraded significantly (*p* < 0.05) [38].
- Surface Roughness: Wang et al. reported additively manufactured zirconia FDPs had higher surface roughness than milled ones but better marginal quality [43].

### 3.7. Outcome

- Implant-Supported Prostheses: Presotto et al. demonstrated SLM-printed Co-Cr frameworks had superior marginal fit (8.4 ± 3.2 µm) and lower stress concentrations vs. milling or casting (*p* < 0.05) [47]. Abu Ghofa and Önöral observed SLM frameworks had the lowest vertical discrepancies (74.2 ± 20.5 µm), outperforming conventional techniques (137.5 ± 18.9 µm, *p* < 0.001) [45].
- Removable Prostheses: Wemken et al. noted milled dentures had the highest trueness, while SLA-printed bases stabilized after microwave sterilization [48]. Neena et al. identified 0.10 mm denture base-tooth offset (DTO) as optimal for 3D-printed denture tooth accuracy (*p* < 0.001) [49].

## 4. Discussion—Navigating the Evolving Landscape of Additive and Subtractive Prosthodontics

The rapid evolution of digital technologies in prosthodontics has ushered in an era of unprecedented possibilities but also complex choices. This scoping review reveals a nuanced reality where neither additive nor subtractive manufacturing emerges as universally superior; rather, each technology carves out distinct niches based on clinical requirements, material properties, and workflow considerations. The evidence identification process for this scoping review employed a rigorous, multi-stage screening methodology to ensure both comprehensive coverage of the relevant literature and methodological precision. By synthesizing findings from 28 core studies and integrating insights from the broader literature, a comprehensive narrative was constructed that addressed the investigative aims while charting a course for future innovation.

### 4.1. The Accuracy Quest: Precision and Adaptability

The enduring strength of subtractive manufacturing lies in its mechanical precision. This paper’s findings corroborate global trends presented by Ainoosah et al. showing milled zirconia crowns consistently achieving marginal gaps below 50 µm, a benchmark rarely matched by 3D-printed counterparts [50]. This aligns with Packaeser et al.’s findings and other biomechanical studies demonstrating that the isotropic properties of milled materials better withstand occlusal forces in posterior regions [51]. The quest for accuracy, long dominated by milling’s deterministic material removal, is now challenged by 3D printing’s layer-by-layer precision. Morón-Conejo et al. demonstrated that DLP-printed interim crowns with optimized postprocessing (50 µm layers, ultrasonic cleaning) rivaled milling’s marginal fit (123.87 ± 67.42 µm vs. 123.89 ± 56.89 µm, *p* = 0.760), aligning with Kumar et al.’s findings of 3D printing’s superior occlusal and axial precision [33,34]. However, Cameron et al. cautioned that milled denture bases consistently outperformed 3D-printed counterparts in trueness, a sentiment echoed by Graf et al., who attributed milling’s edge to reduced anisotropic shrinkage and material homogeneity [35,36,37]. Nevertheless, the narrative grows more intriguing when examining complex geometries. Recent breakthroughs in volumetric 3D printing have enabled printed lattice structures for partial denture frameworks that surpass milled versions in tissue compatibility while maintaining clinically acceptable fit [52,53].

### 4.2. Efficiency Reimagined: Beyond Print Speed

Efficiency, however, tilts toward additive workflows. Song et al. highlighted 90° build angles in minimizing material waste, albeit at the cost of prolonged printing times, while Casucci et al. reported 30% shorter chairside times for digital dentures [32,46]. Yet, subtractive methods retain advantages in high-throughput settings, particularly for monolithic zirconia restorations, where milling avoids the post-curing complexities of printed ceramics [43].

Traditional efficiency metrics favoring milling’s faster production times tell only part of the story [34]. Notably, 3D printing enables distributed manufacturing models where designs are transmitted digitally and printed locally, potentially revolutionizing care delivery in remote areas [54]. As per Dawood et al., 3D printing combined with CAD and digital scanning allows for the efficient and precise fabrication of complex prosthodontic restorations, including metal-based prostheses and lost-wax patterns. While the technology enhances workflow efficiency, factors such as material costs, post-processing requirements, and operator expertise must be accounted for [55]. Joda et al. found that fully digital workflows were faster and more cost-effective for zirconia iFDPs, though minor adjustments were sometimes needed, highlighting the importance of workflow selection and technician skill [56].

Material efficiency introduces another compelling advantage. At first glance, powder-based 3D printing appears far superior, achieving near-complete material reuse, whereas traditional milling often wastes 30–90% of raw material. However, researchers have uncovered promising solutions to milling’s inefficiencies. For instance, Ding et al. demonstrated that dental CAD/CAM zirconia waste can be effectively recycled through a straightforward method of pre-sintering the residuals at 950–1000 °C [57]. This breakthrough not only mitigates waste but also enhances the sustainability of subtractive manufacturing, a considerable advantage as environmental awareness grows within healthcare. As Duane et al. highlight, the rising emphasis on eco-conscious practices demands proper recycling protocols to avoid unintended ecological trade-offs [58].

### 4.3. The Material Science Frontier

Material behavior remains a decisive factor. Gad et al. underscored milled resins’ superior color stability (ΔE00 < 1.5) and dimensional resilience after aging, contrasting with 3D-printed resins’ susceptibility to hygroscopic expansion [38]. For metals, Presotto et al. found SLM-printed Co-Cr frameworks achieved marginal fits (8.4 ± 3.2 µm), surpassing casting and milling [47], while Abu Ghofa and Önöral noted SLM’s vertical discrepancies (74.2 ± 20.5 µm) were 45% lower than conventional techniques [45]. These advances, however, are tempered by challenges: Wang et al. reported higher surface roughness in 3D-printed zirconia FDPs, which may compromise polishability and biofilm resistance, corroborated by Hassanpour et al.’s 2024 analysis of post-processing parameters on 3D-printed dental appliances [43,59].

Emerging hybrid materials, such as fiber-reinforced polymers and bioactive ceramics, offer promising solutions to bridge these gaps. Furthermore, next-generation self-healing materials demonstrate significant potential in mitigating microcrack propagation within 3D-printed dentures, as reported by recent studies [60,61]. Such innovations could merge additive manufacturing’s design flexibility with milling’s structural integrity, particularly for implant-supported prostheses. Particularly promising are stimuli-responsive 4D printing materials that adapt their properties post-insertion, such as temperature-modulated gingival masks that improve denture seal over time [62,63].

Conversely, subtractive manufacturing is not standing still. The development of self-healing CAD/CAM milling blocks containing microencapsulated repair agents may mitigate milling’s vulnerability to microcracks [64]. For removable prostheses, antimicrobial milled polymers incorporating silver nanoparticles offer infection control benefits that current 3D printing materials cannot replicate [65].

### 4.4. Clinical Translation: Bridging the Digital Divide

Clinically, subtractive methods dominate implantology and fixed prosthodontics due to their proven longevity. Lee et al.’s 2.0 mm hollow pontic design for 3D-printed FDPs and Kim et al.’s SLA-printed Co-Cr copings (70.2 ± 15.5 µm discrepancies) exemplify additive manufacturing’s growing viability, yet their adoption hinges on long-term fatigue data [36,42]. For removable prostheses, Wemken et al. affirmed milled dentures’ superior trueness but noted SLA-printed bases’ stability post-sterilization, suggesting niche applications in edentulous patients requiring frequent adjustments [48].

Patient-centric outcomes, such as masticatory efficiency and comfort, remain understudied. Casucci et al. found no significant differences in bite force between digital and conventional dentures, yet broader studies, including Fouda et al.’s 2024 systematic review on patient perceptions, reveal higher patient satisfaction with milled dentures due to superior occlusal stability [32,66].

The translation of laboratory precision to clinical success remains fraught with challenges. Digital workflows reduce appointment times, as Casucci et al. presented in 2025 [32], but also demand new skill sets that are not yet standardized in dental curricula. While Fernandez et al., in 2015, found limited CAD/CAM adoption in dental education (≤10% of cases) despite widespread awareness, with program directors driving early curricular integration (50% vs. 12%), a more recent study by Porcherot et al. in 2023 demonstrates tangible educational progress: digital training now enhances conventional denture fabrication skills, and hybrid assessment methods effectively evaluate holistic competencies, marking a shift from tentative planning to proven pedagogical integration [67,68]. The proliferation of chairside 3D printing systems (≤USD 20,000) is democratizing access (Revilla-León et al., 2023), yet quality control remains inconsistent without standardized post-processing protocols [59,69].

### 4.5. Future Directions: A Protocol for Innovation

The path forward lies in harmonizing additive and subtractive workflows. Hybrid approaches, such as milling 3D-printed blanks for high-precision margins, could exploit both technologies’ strengths. Katheng et al.’s 30 min, 40 °C postpolymerization protocol for SLA dentures and Metin et al.’s 30–45° build angles for optimal trueness exemplify the need for standardized protocols. Artificial intelligence (AI)-driven design algorithms, though beyond the scope of these studies, could further optimize support structures and minimize anisotropic distortion.

1. Intelligent Manufacturing. Machine learning algorithms that predict and compensate for material shrinkage patterns could eliminate 3D printing’s accuracy disadvantages [70]. Similarly, AI-driven nesting software can optimize milling strategies to reduce waste by 30–40%.
2. Convergence Technologies. The most exciting developments lie at the additive–subtractive interface. Systems like hybrid multi-tasking platforms use milling for critical fit surfaces while 3D printing tissue-facing contours, achieving unprecedented precision–efficiency balances.
3. Material science must prioritize bioactive and self-healing resins to enhance 3D printing’s clinical relevance.
4. Regulatory Framework Development. As 3D-printed dentures enter mainstream care, standardized post-market surveillance systems must track long-term performance. This requires collaboration between manufacturers, clinicians, and regulatory bodies.

### 4.6. Limitations of This Scoping Review

While this scoping review provides a comprehensive synthesis of current evidence, several limitations must be acknowledged alongside its methodological strengths. First, the predominance of in vitro studies (27/28) limits extrapolation to clinical outcomes, as aging, occlusal forces, and intraoral humidity were simulated rather than replicated. Second, heterogeneity in measurement protocols, e.g., Morón-Conejo et al.’s replica technique vs. Camargo et al.’s micro-CT analysis, precludes direct meta-analytic comparisons. Third, the exclusion of non-English studies and gray literature may introduce publication bias. Finally, rapid technological evolution outpaces peer-reviewed validation, necessitating continual updates to clinical guidelines.

This balanced appraisal underscores that while the review provides robust guidance for technology selection, clinicians should interpret findings within their specific practice constraints and patient populations. The limitations primarily reflect gaps in current research rather than methodological flaws, suggesting where future primary studies should focus.

## 5. Conclusions: A Symbiotic Future for Prosthodontic Innovation

The prosthodontic landscape is evolving beyond the “either/or” dichotomy, embracing a “yes, and” paradigm, where additive and subtractive technologies complement rather than compete.

Key Takeaways

1. Milling and 3D Printing Are Complementary:
  - Use milling for high-precision, high-stress cases.
  - Use 3D printing for complex designs, patient-specific solutions, and interim prostheses.
2. Choose Based on Patient Needs, Not Technology
  - Durability? => Milling.
  - Customization or complex anatomy? => 3D printing.
  - Workflow optimization and material selection are paramount.
3. Future Focus
  - Hybrid workflows will dominate, combining both technologies for optimal results.
  - Research ought to prioritize material advancements, AI integration, and long-term clinical data.

The studies reviewed here provide not only answers but, more importantly, the right questions to guide this transformative journey. The path forward lies in harmonizing the strengths of both methods, ensuring that prosthodontics continues to evolve as both a science and an art, tailored to the ever-changing needs of patients.

## Figures and Tables

**Table 1:** Eligibility criteria with rationale.

Category	Inclusion Criteria	Exclusion Criteria	Rationale
Study Design	Comparative studies (in vitro, in vivo, clinical trials); systematic reviews (background only)	Non-comparative studies; opinion papers; editorials; conference abstracts without full data	Ensures comparison of methods/technologies; excludes low-evidence sources
Interventions	Technology comparison, 3D printing and CAD/CAM milling in prosthodontics	Studies with no comparison of technology	Addresses the core research question of technology performance
Outcomes	Quantitative data on	Qualitative outcomes	Focuses on measurable, clinically relevant parameters for objective analysis
	Accuracy (marginal gap, trueness),	Irrelevant metrics	
	Efficiency (time, cost)	(e.g., patient satisfaction alone).	
Applications	Fixed and Removable Prosthesis on Teeth and Implants	Non-prosthodontic uses (e.g., orthodontics, endodontics)	Maintains focus on prosthodontic applications
Publication	Peer-reviewed articles (2015–Feb 2025)	Non-peer-reviewed (theses, patents);	Ensures methodological rigor and relevance to current digital workflows.
	English language	Non-English (without translation); pre-2015	

**Table 2:** Inclusion of heterogeneous studies.

Study Type	Extraction Focus
In vitro (n = 27)	Accuracy (µm), material properties, efficiency
Retrospective (n = 1)	Long-term performance data

## Data Availability

No new data were created.

## Supplementary Materials

The following supporting information can be downloaded at: https://www.mdpi.com/article/10.3390/jcm14144837/s1. Supplementary File S1: Search strategy; Supplementary File S2: Completed PRISMA-ScR Checklist.

## Abbreviations

The following abbreviations are used in this manuscript:

CAD/CAM	Computer-Aided Design/Computer-Aided Manufacturing
FDP	Fixed Dental Prostheses
RMSE	Root Mean Square Error
PMMA	Polymethyl Methacrylate
SLA	Stereolithography
DLP	Digital Light Processing
SLM	Selective Laser Melting
Co-Cr frameworks	Cobalt-Chromium frameworks
DTO	Denture Base-Tooth Offset
iFDP	Implant-Fixed Dental Prosthesis
SR100	SprintRay 100-µm group
