# Supplementary material for: Three-Dimensional Printing and CAD/CAM Milling in Prosthodontics: A Scoping Review of Key Metrics Towards Future Perspectives
Source: J Clin Med. 2025 Jul 8;14(14):4837. doi: 10.3390/jcm14144837 (PMC12294912; doi:10.3390/jcm14144837)
Supplement: Supplementary file 1 [file jcm-14-04837-s001.zip › jcm-3687457-supplementary/Supplementary File S1.pdf]

Supplementary File S1

Keywords and Subject Headings Used During the Search

**Database**	**Search strategy**
PubMed accessed March 28, 2025	(("3D printing"[Title/Abstract] OR "additive manufacturing"[Title/Abstract] OR "stereolithography"[Title/Abstract] OR "digital light processing"[Title/Abstract] OR "DLP"[Title/Abstract] OR "FDM"[Title/Abstract])<br>AND<br>("CAD/CAM"[Title/Abstract] OR "computer-aided design"[Title/Abstract] OR "subtractive manufacturing"[Title/Abstract] OR "milling"[Title/Abstract] OR "CNC machining"[Title/Abstract])<br>AND<br>("prosthodontics"[MeSH] OR "dental prosthesis"[Title/Abstract] OR "crown"[Title/Abstract] OR "bridge"[Title/Abstract] OR "denture"[Title/Abstract] OR "implant prosthesis"[Title/Abstract])<br>AND<br>("accuracy"[Title/Abstract] OR "trueness"[Title/Abstract] OR "precision"[Title/Abstract] OR "marginal fit"[Title/Abstract] OR "efficiency"[Title/Abstract] OR "workflow"[Title/Abstract] OR "material properties"[Title/Abstract])<br>)<br>AND<br>("2015/01/01"[Date - Publication] : "2025/02/28"[Date - Publication])
Scopus accessed March 28, 2025	TITLE-ABS-KEY (("3D printing" OR "additive manufacturing" OR "stereolithography" OR "digital light processing" OR "DLP" OR "FDM")<br>AND<br>("CAD/CAM" OR "computer-aided design" OR "subtractive manufacturing" OR "milling" OR "CNC machining")<br>AND<br>("prosthodontics" OR "dental prosthesis" OR "crown" OR "bridge" OR "denture" OR "implant prosthesis")<br>AND<br>("accuracy" OR "trueness" OR "precision" OR "marginal fit" OR "efficiency" OR "workflow" OR "material properties")<br>)<br>AND<br>PUBYEAR > 2014 AND PUBYEAR < 2025
Web Of Science accessed March 28, 2025	TS=(("3D printing" OR "additive manufacturing" OR "stereolithography" OR "digital light processing" OR "DLP" OR "FDM")<br>AND<br>("CAD/CAM" OR "computer-aided design" OR "subtractive manufacturing" OR "milling" OR "CNC machining")<br>AND<br>("prosthodontics" OR "dental prosthesis" OR "crown" OR

	"bridge" OR "denture" OR "implant prosthesis")<br>AND<br>("accuracy" OR "trueness" OR "precision" OR "marginal fit" OR "efficiency" OR "workflow" OR "material properties")<br>)<br>AND<br>PY=(2015-2025)
Embase accessed March 28, 2025	1. 'prosthodontics'/exp OR ('dental prosthesis' OR 'crown' OR 'bridge' OR 'denture' OR 'implant prosthesis'):ti,ab<br>2. ('3D printing' OR 'additive manufacturing' OR 'stereolithography' OR 'digital light processing' OR 'DLP' OR 'FDM'):ti,ab<br>3. ('CAD/CAM' OR 'computer-aided design' OR 'subtractive manufacturing' OR 'milling' OR 'CNC machining'):ti,ab<br>4. ('accuracy' OR 'trueness' OR 'precision' OR 'marginal fit' OR 'efficiency' OR 'workflow' OR 'material properties'):ti,ab<br>5. 1 AND 2 AND 3 AND 4<br>6. [2015-2025]/py AND [english]/lim
